# Engineered *Saccharomyces cerevisiae* for lignocellulosic valorization: a review and perspectives on bioethanol production

**DOI:** 10.1080/21655979.2020.1801178

**Published:** 2020-08-15

**Authors:** Joana T. Cunha, Pedro O. Soares, Sara L. Baptista, Carlos E. Costa, Lucília Domingues

**Affiliations:** CEB – Centre of Biological Engineering, University of Minho, Campus Gualtar, Braga, Portugal

**Keywords:** *Saccharomyces cerevisiae*, genetic engineering, 2^nd^ generation bioethanol, biorefineries, lignocellulosic biomass

## Abstract

The biorefinery concept, consisting in using renewable biomass with economical and energy goals, appeared in response to the ongoing exhaustion of fossil reserves. Bioethanol is the most prominent biofuel and has been considered one of the top chemicals to be obtained from biomass. *Saccharomyces cerevisiae*, the preferred microorganism for ethanol production, has been the target of extensive genetic modifications to improve the production of this alcohol from renewable biomasses. Additionally, *S. cerevisiae* strains from harsh industrial environments have been exploited due to their robust traits and improved fermentative capacity. Nevertheless, there is still not an optimized strain capable of turning second generation bioprocesses economically viable. Considering this, and aiming to facilitate and guide the future development of effective *S. cerevisiae* strains, this work reviews genetic engineering strategies envisioning improvements in ^2^nd^^ generation bioethanol production, with special focus in process-related traits, xylose consumption, and consolidated bioprocessing. Altogether, the genetic toolbox described proves *S. cerevisiae* to be a key microorganism for the establishment of a bioeconomy, not only for the production of lignocellulosic bioethanol, but also having potential as a cell factory platform for overall valorization of renewable biomasses.

## GRAPHICAL ABSTRACTS

**Figure:**
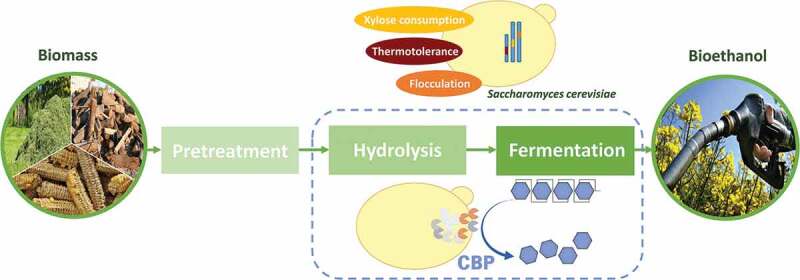

## 1. Introduction

With the depletion of fossil fuel reserves, the world faces a demand for renewable energy sources for the production of biofuels and value-added products. The growing consumption of fossil fuels has anticipated the reserves exhaustion for the next 40–50 years [1]. This rapid consumption of fossil fuels also intensified the emission of greenhouse gas and all the climate changes promoted by global warming [2]. A key step for the development of sustainable processes is the shift from petroleum- to bio-based processes in a biorefinery context, defined as ‘the sustainable processing of biomass into a spectrum of marketable products (food, feed, materials, chemicals) and energy (fuels, power, heat)’ [3].

Lignocellulose is one of the most abundant renewable biomass sources available on Earth with the advantage of not competing with land for food production [4,5]. Lignocellulosic biomass can be obtained from energy crops, aquatic plants, forest biomass and wastes and agricultural residues [6,7]. The complex and recalcitrant structure of lignocellulosic biomass comprises cellulose, hemicellulose and lignin. The content of each fraction and the structural arrangement between those fractions may vary with the source of the biomass, and with that, the accessibility to monomer sugars will also differ [8]. Lignin is a complex and highly branched polyphenolic polymer mainly present in the cell wall of hard- and softwoods, providing rigidity to the plants. The cellulosic and hemicellulosic fractions, comprise the main carbon sources for the production biofuels and value-added products and constitute up to two thirds of lignocellulosic biomass [9].Cellulose is a homopolymer of D-glucose and can represent up to 70% of the total lignocellulosic biomass [10]. Its crystalline matrix structure, due to the extensive hydrogen bonds between glucose molecules, makes it resistant to de-polymerization and insoluble in water [11].

On the other hand, hemicellulose is a heteropolymer of short, linear, and branched chains of several monomers, including hexoses (glucose, galactose, and mannose) and pentoses (xylose and arabinose). The backbone of hemicellulose is mainly composed of xylan (β-1,4-linked xylose residues), which may represent up to 50% of the composition in some tissues of grasses and cereals [9].Production of second generation bioethanol requires the following main steps: (1) pretreatment to break the recalcitrant structure of lignocellulose, (2) hydrolysis of cellulose and hemicellulose to fermentable sugars, (3) microbial fermentation for the production of ethanol [12]. Pretreatment of lignocellulosic biomass is required to disrupt lignin-cellulose-hemicellulose complexes, which results in the removal of lignin, decrease of the cellulose crystallinity and increase of the surface area and porosity of the biomass for accessibility of the hydrolytic enzymes. This step includes acid-based, hydrothermal, chemical, and oxidative methods or the use of solvents, and often results in the production of inhibitory compounds [8,13–15]. These lignocellulosic-derived by-products generated in pretreatment process act as inhibitors for enzymes and microorganisms when their concentration is above a critical threshold. These inhibitors include sugar acids, acetic acid, formic acid, levulinic acid, hydroxymethylfurfural (HMF) and furfural [16].

In order to obtain fermentable sugars, the solid (cellulose) and liquid (hemicellulose) fractions resulting of pretreatment should be submitted to a hydrolysis process, normally performed by the addition of acid catalysts or enzymes. The major drawbacks when applying acid hydrolysis are the production of inhibitors through degradation of sugars and the recovery or neutralization of the acids prior to the fermentation process [8,9]. Enzyme specificity to the substrate, low temperatures and generation of minimum inhibitors are the key aspects of enzymatic hydrolysis that render this process as the most promising and effective. On the other hand, enzymes costs and yields lower than theoretical values are the main holdups associated with enzymatic hydrolysis [8,17].

Taking into account the bottlenecks associated with pretreatment and hydrolysis processes, a cost-efficient exploitation of lignocellulosic biomass for biofuels and value-added products is dependent on a robust microorganism to perform the fermentation process. *Saccharomyces cerevisiae*, a yeast generally regarded as safe (GRAS), has been broadly used in the biotechnology industry. It is used in large-scale operations and is a model eukaryotic system, with an in-depth studied molecular and cellular biology and a variety of genetic tools available. As an eukaryotic organism, it has multiple organelles that could be used as compartments for the biosynthesis of different compounds. *S. cerevisiae* is widely used in industry, exhibiting high tolerance against harsh industrial conditions [18,19]. Together, these characteristics have triggered the development of *S. cerevisiae* as a chassis microorganism for metabolic engineering aiming the valorization of lignocellulosic biomass [5]. Furthermore, industrial environments have been recognized as a source of *S. cerevisiae* strains with higher robustness, fermentation capacity and resistance to stress factors found in harsh industrial processes when compared with laboratory strains [20]. Thermotolerance is one of the traits presented by some industrial yeast strains that can be desirable for a consolidated process of lignocellulose valorization due to the higher optimal temperatures of hydrolytic enzymes in comparison with the optimal temperature for *S. cerevisiae* fermentation [21–23]. Industrial isolates may also possess intrinsic capabilities and specificities to respond to genetic engineering, either for tolerance or pentose metabolism, which reveals the necessity of a personalized genetic engineering to the selected yeast chassis and lignocellulosic biomass used in the fermentation process [21,24,25].

Bearing in mind both the energetic and economical goals of the biorefinery concept, i.e., replace the use of petroleum by renewable carbons for fuel production as well as establish a robust biobased economy, it is crucial to increase the efficiency and economic return of biofuel plants. In fact, bioethanol was considered one of the top value chemicals to be obtained from biomass [26] and, besides its value as a biofuel, its importance for the formulation of hydro-alcoholic gel, sanitizers and other disinfectants gained increased attention in response to the 2020 Covid-19 pandemic. Actually, several industries announced commercial-scale lignocellulosic ethanol plants, with the majority using *S. cerevisiae* for the fermentation step, however the global volumetric production of this second generation bioethanol is still less than 1% of that of 1^st^ generation processes [27]. While industries try to maintain secrecy regarding the hurdles preventing the intensification of their processes, it is known that, besides non-yeast-related problems (such as seasonal and regional fluctuations in lignocellulosic biomass production, or the presence of non-plant high density solids), the fermentative step is a very challenging part of the overall process: (1) the yeast strains require constant modifications to cope with the continued optimization of the upstream unit operations (e.g., pretreatment and hydrolysis technology); (2) there is a necessity for highly thermotolerant strains, not only to favor a consolidated bioprocess, but also to decrease the regional-dependent cooling costs and (3) bacterial contamination is more problematic than in 1^st^ generation processes (where the inhibitory composition of lignocellulosic-pretreated media hampers yeast growth and also lower concentrations of the bacterial-inhibitor ethanol are obtained), which requires the development of more highly robust and tolerant yeast strains.

Considering this constant need for development and optimization of robust and process-optimized microorganisms, we provide an overview of genetic engineering strategies previously applied to *S. cerevisiae* to improve the conversion of lignocellulosic biomass into ethanol, with special focus in the optimization of process-related traits, valorization of lignocellulose through xylose consumption and decrease of hydrolysis associated cost by development of consolidated bioprocessing strains.

## 2. Improvement of process-related traits

### 2.1. Thermotolerance

High-temperature fermentation technology is expected to reduce the cost of bioconversion of biomass to fuels or chemicals. Ethanol production from lignocellulosic biomass can be achieved through a process known as separate hydrolysis and fermentation (SHF), however, this process has a major drawback associated with feedback inhibition of hydrolytic enzymes due to the accumulation of sugar monomers. To overcome this disadvantage simultaneous saccharification and fermentation (SSF) or simultaneous saccharification and co-fermentation (SSCF, when both hexose and pentose sugars are fermented) can be performed for ethanol production [8]. However, the difference between the optimal temperatures of hydrolytic enzymes (45–50°C) and the optimal growth temperature of *S. cerevisiae* (30°C) turned thermotolerance in an attractive feature for yeasts used for ethanol production from lignocellulosic biomass [21,22]. Thermotolerance influence in ethanol production from lignocellulosic biomass was indeed demonstrated in a recent study with industrial *S. cerevisiae* strains, where a more thermotolerant strain was more efficient in fermenting an Eucalyptus globulus hydrolyzate, presenting faster xylose consumption and higher ethanol production [21].

Thermotolerant yeast can ferment at temperatures above 40°C. In fact, some yeast, such as *Kluyveromyces marxianus* or *Ogataea polymorpha*, naturally possess this capacity (with the mechanism behind their superior tolerance being not yet elucidated) [28]. Nevertheless, the ethanol yields of these strains are still far from the theoretical, and their metabolic toolbox, while growing due to their potential industrial application, are still underdeveloped in comparison with *S. cerevisiae* [28].Therefore, the screening and isolation of *S. cerevisiae* strains with improved fermentation ability under high temperatures have been pursued [22,29]. Recently, a growth phenotypic screening of 12 industrial *Saccharomyces* strains was conducted and the most thermotolerant strain selected [29]. The physiological characterization at 39°C and 30°C, in well-controlled bioreactors, of the selected thermotolerant strain and the control strain CEN.PK113-7D, revealed that increased temperature tolerance coincided with higher energetic efficiency of cell growth [29]. Thus, temperature intolerance is a result of energy-wasting processes, such as increased turnover of cellular components due to temperature-induced damage, like protein misfolding [29]. Accordingly, high-temperature tolerance in yeast cells involves the activation and regulation of specific stress-related genes, which involves the synthesis of specific compounds such as heat shock proteins (HSPs) to protect the organism from high temperature stress. Trehalose is another protective compound accumulated under high temperatures, as it helps the stabilization of cytoplasmatic membrane and cellular proteins [30,31].

Recently, the differential proteomic responses of three distinct *S. cerevisiae* strains, an industrial wine strain, ADY5, a laboratory strain, CEN.PK113-7D and an industrial bioethanol strain, Ethanol Red, grown at sub- and supra-optimal temperatures were studied under chemostat conditions, mimicking the industrial processes [29,32]. The proteomic profile of these strains was performed by SWATH-MS, allowing the quantification of 997 proteins [32]. Overall, proteomic data evidenced that at high temperature (39°C), the amino acid biosynthetic pathways and metabolism represent the main function recruited [32]. The variability of responses of the three strains examined showed that no general rules can be assumed for different *S. cerevisiae* strains, and that the temperature-response is highly dependent on their genetic and environmental background [32]. At 39°C, the best performing strain at supra-optimal temperatures, increased the expression of proteins involved in ergosterol and glycogen synthesis, along with Hsp104p, which are known to play a crucial role in heat adaptation [32].

In spite of being a multi-trait phenotype highly dependent on the strain genetic background, some mutation and genetic engineering strategies have been successfully employed for enhancing yeast thermotolerance. Kim et al (2011) identified 8 genes responsible for ethanol and heat tolerance using transposon mutagenesis that randomly disrupt or affect the transcription of genes [33]. After exposure to ethanol, the selected ethanol-tolerant mutants were exposed to 42°C, allowing the isolation of two strains simultaneously tolerant to ethanol and temperature. One of those strains presented down-regulation of *SSK2* (encoding a MAP kinase kinase kinase of HOG1 mitogen-activated signaling pathway) and*PPG1* (encoding a Protein Phosphatase involved in Glycogen accumulation), while the other has *PAM1* gene(which encodes an essential protein of unknown function) knocked-out. The plasmid-mediated expression of those genes reverted the ethanol and heat tolerance, suggesting that repression of those genes might be the mechanism for ethanol and thermotolerance [33].

Liu et al. (2014) expressed heat shock genes from *Thermoanaerobacter tengcongensis* to develop a thermotolerant *S. cerevisiae* strain [34]. Amongst the ten genes cloned into *S. cerevisiae*, strains harboring *tte2469, gros2* and *ibpa*(encoding ubiquitin, HSP10 and HSP20, respectively)presented a superior cell viability compared to parental strain when grown at 42°C or exposed to a temperature gradient from 35 to 45°C. The authors referred that *gros2* and *ibpa*, codifying for small HSPs, may have a dual protective role in yeast cells, as they prevent protein denaturation, misfolding, and aggregation and are indirectly involved in increased expression of stress-response genes (*cdc19*- which encodes a pyruvate kinase involved in ATP production; and *tps1-* encoding trehalose-6-phosphate synthase), promoting a synergetic effect between HSPs, trehalose, and energy-generating pathway to withstand heat stress [34].

Shahsavarani et al. (2012), overexpressed a new allele of *RSP5* with higher transcriptional levels, *RSP5-C*, to develop a higher thermotolerant strain [35]. *RSP5* encodes for an E3 ubiquitin, involved in the ubiquitination of proteins, regulating the trafficking and eventual degradation of proteins in various cellular compartments [36]. It is also associated to DNA repair and RNA transport [37]. The overexpression of *RSP5* in a non-thermotolerant *S. cerevisiae* strain conferred thermotolerance at 41°C, while the overexpression of *RPS5-C* allele in thermotolerant strain raised its upper limit of heat tolerance to 43°C. Besides the acquisition or stronger heat tolerance phenotype associated to *RSP5-C*, it was shown that strains with this allele also displayed increased cell-wall stability, ethanol and osmotic stress tolerance, important features in a strain for bioethanol production [35].

In a different approach, the deletion of *Dfg5*, encoding a glycosylphosphatidylinositol (GPI)-anchored plasma membrane protein, enhanced heat tolerance in *S. cerevisiae*. Strains with deleted *Dfg5* exhibited higher heat tolerance after exposure to 41°C, as well as decreased membrane permeability and lower levels of reactive oxygen species (ROS). The transcriptional analysis of *dfg5∆* mutants revealed the up-regulation of 14 genes involved in stress response and detoxification and the down-regulation of 13 genes. From this analysis, the authors suggested that Dgf5 regulates the expression of heat tolerance genes (HTGs), as GPI-anchored proteins have been associated with transcriptional regulation or signal transduction. Also they proposed a model where, in the absence of Dgf5, heat sensors in the yeast cell wall regulate the expression of HTGs, enhancing tolerance in the mutant yeast [38].

Khatun et al. (2017) applied a completely different strategy through the use of artificial zinc finger proteins (AZFP) to improve thermotolerance in *S. cerevisiae*. AZFP are synthetized transcription factors based on zinc finger proteins used in the development of desired phenotypes through metabolic reprogramming of microorganisms [39]. AZFP libraries contain AZFPs with random zinc motifs that carry a DNA binding domain that activates or represses gene transcription [40,41]. The authors applied this strategy in a *S. cerevisiae* strain used in the consolidated bioprocessing (CBP) of Jerusalem artichoke stalk (JAS) for ethanol production. The engineered strain MNII-AZFP displayed higher thermotolerance compared to the parental strain, as this strain could optimally grow at 42°C and had higher cell viability upon exposure to 50°C. As observed in other thermotolerant yeast, trehalose accumulation increased and ROS levels were lowered. Heat shock proteins, signaling factors and proteins involved in vacuole transport and targeting, presented increased transcription levels when exposed to high temperature [39].

Satomura et al (2016) performed heat adaptation experiments with a non-thermotolerant *S. cerevisiae* strain, obtaining a strain with thermotolerance at 38°C that presented accumulation of trehalose. The intermediate strains during the adaptive process were collected and subjected to whole-genome analysis, allowing the identification of one-point mutations in the *CDC25* gene [42]. Cdc25p is a guanine nucleotide exchange factor involved in the regulation of intracellular cAMP levels, and thus, in the cAMP-dependent protein kinase (PKA) signaling pathway [43]. The cAMP-PKA pathway is responsible for the inactivation of the Msn2p/Msn4p transcriptional activators that are responsible for general stress responses in *S. cerevisiae*. Mutations in Cdp25p resulted in lower levels of cAMP under high-temperature conditions, which in turn lead to the activation of Msn2p/Msn4p by cAMP-PKA signaling pathway. The authors confirmed the upregulation genes involved in stress response and organelle membrane by Msn2p/Msn4p, suggesting that one-point mutations in*CDC25* resulted in the acquisition of the thermotolerance phenotype. *CDC25* mutants also displayed efficient ethanol fermentation under heat stress [42].

### 2.2. Flocculation

Another important feature in microorganisms used for bioethanol production is flocculation. Flocculant yeast have the ability to form multicellular clumps and settle quickly from fermentation broth which facilitates a cost-efficient biomass recovery with a considerable reduction of energy costs [44,45]. Flocculation allows an easily retention and immobilization of yeast cells within fermenters, resulting in high cell density [46] and decreasing the risk of contamination [47]. Moreover, continuous operation at higher dilution rate than the maximum growth rate is accomplished [48–51], increasing ethanol productivity and making ethanol production more economically competitive [48,49]. Also, flocculation is pointed as a beneficial trait in second-generation bioethanol production [52] as it enhances cell protection toward inhibitory hydrolyzates [53,54] besides ethanol [55,56].

Yeast flocculation is a non-sexual aggregation of cells, a reversible process dependent on calcium, where lectins play a major role. These cell wall proteins recognize and adhere to mannose residues of the cell wall of the surrounding yeast cells, promoting aggregation [57,58]. Depending on sugar sensitivity, flocculation can be divided in the Flo1 type, inhibited by mannose, and the NewFlo type, inhibited by both mannose and glucose. Due to the high concentrations of glucose used in bioethanol production, Flo1 is the flocculation preferred phenotype for this process [46,58,59].

Flocculation in yeast cells is majorly controlled by genetic factors. *FLO1, FLO5, FLO9,* and *FLO10* were identified in *S. cerevisiae* as the four dominant genes in flocculating strains [60,61]. *FLO1* is the most studied gene amongst the four dominant genes regarding flocculation, as its introduction in a non-flocculating *S. cerevisiae* strain resulted in a flocculation phenotype [46,62]. Watari et al. (1994), introduced two variations of *FLO1* in non-flocculating brewing and other industrial yeasts. Both the intact version of *FLO1* present in *S. cerevisiae* chromosomal DNA (*FLO1L*) and a smaller version (*FLO1S*, lacking 675 amino acids in a highly repeated region of the open reading frame) were used, and while both induced flocculation, *FLO1L* conferred a stronger flocculant phenotype. The authors also integrated the *FLO1L* into the *ADH1* locus, resulting in a stable flocculant strain after 100 generations important for industrial processes [63]. A more recent study with a high flocculant *S. cerevisiae* strain showed that *FLO5* plays an important role in cell-surface adhesion. In fact, the deletion of *FLO5* caused a strong reduction in flocculation capacity when compared with the deletion of *FLO1* [64].

Govender et al. (2008) suggested a strategy for the optimization of flocculation through the controlled expression of *FLO1, FLO5,* and *FLO11* in *S. cerevisiae* [65]. Introduction of flocculation genes in yeast can affect their fermentation performance, and to overcome this the authors elaborated a strategy in which the native promotors of *FLO1, FLO5* and *FLO11* were replaced with the inducible promotors *ADH2* and *HSP30. ADH2* promotor is repressed during growth on glucose and is activated in the transition to grow on ethanol [66,67]. *HSP30* is induced in the stationary phase of growth, coinciding with the depletion of glucose and is also activated by stress factor, such as heat shock or exposure to ethanol [68,69]. The study confirmed that *FLO1* and *FLO5* expression under *HSP30* promotor occurred upon depletion of glucose and in response to heat shock and ethanol exposure, and after glucose depletion under *ADH2* promotor. It was also observed that *FLO1* and *FLO5* induced flocculation was stronger under regulation of *ADH2* promotor when compared to *HSP30* promotor. Furthermore, the *FLO1* mutants exhibited a stronger flocculant phenotype when compared to *FLO5* mutants. Regarding *FLO11*, which induces invasive growth and flor formation, as transcription of this gene is repressed by glucose, *ADH2,* and *HSP30*-mediated expression were not effective. However, this is an interesting strategy, especially with *ADH2*-mediated expression of flocculant genes, as the regulated induction of flocculant phenotype could be applied to optimize bioethanol production processes, facilitating downstream processing without compromise production performance [65].

## 3. Valorization of the hemicellulosic fraction of lignocellulose

### 3.1. Xylose consumption

A viable production of bioethanol involves the efficient conversion of the hemicellulosic fraction, mainly composed by xylose, into ethanol. Many yeasts have in their genome the codifying genes for xylose consumption and metabolization. Still, some of them cannot grow on xylose, which can be explained by deficient regulation of xylose pathway expression or enzymes [70]. Several xylose-assimilating yeasts have been isolated from different sources, but only a small percentage is capable of producing ethanol from this pentose [71–73]. These naturally xylose-fermenting yeast, such as *Scheffersomyces stipitis* (formerly known as *Pichia stipitis), Candida tropicalis* or *Spathaspora passalidarum* can convert xylose into ethanol, however low tolerance to ethanol and lignocellulosic-derived inhibitors are major drawbacks, as well as strict culture conditions requirements (e.g., pH and dissolved oxygen levels) to maintain the xylose fermentation performance [74,75]. Consequently, taking advantage of the innate capacity of ethanol production of *S. cerevisiae*, the introduction of heterologous xylose assimilation pathways and optimization of internal metabolism through metabolic engineering have been applied to obtain *S. cerevisiae* strains that efficiently ferment xylose into ethanol, avoiding the formation of by-products.

Throughout the years, two different pathways have been expressed in *S. cerevisiae* to convert xylose into xylulose: the oxidoreductase and the isomerase pathway. The oxidoreductase pathway is used by xylose-fermenting yeasts and occurs mainly under aerobic conditions. It is composed by two enzymatic reactions catalyzed by xylose reductase (XR) and xylitol dehydrogenase (XDH), converting xylose into xylulose through xylitol, in a two-step redox reaction [76]. This pathway starts with the reduction of xylose into xylitol by XR that preferably uses NADPH as cofactor; then xylitol is oxidized to xylulose by XDH, in a reaction that only uses NAD^+^ as cofactor [77]. Contrary to the oxidoreductase pathway, in the isomerase pathway the conversion of xylose into xylulose is a one-step reaction catalyzed by xylose isomerase (XI), a reaction without cofactor requirement [74,78]. The majority of XIs identified so far come from bacterial strains, however, some anaerobic fungi are also able of assimilating xylose through XI [79–81]. Common to both pathways is the phosphorylation of xylulose into xylulose-5-phosphate by xylulokinase (XK), a gene endogeneous of *S. cerevisiae* but that requires overexpression in order to obtain an efficient consumption of xylose [74].

The first attempts of cloning XI into *S. cerevisiae* failed due to difficulties of expressing functionally bacterial XIs in yeast [82–84]. However, the discover of XI coding genes from anaerobic fungi [80,81,85–88] and bacteria from *Thermus thermophiles* [89], *Clostridium phytophermentans* [90] and *Bacteroides stercoris* [91] allowed the successful expression of functionally XIs and consequently xylose fermentation in *S. cerevisiae*.

Heterologous XIs have been subjected to adaptation, either by codon optimization or directed evolution, to improve expression and activity in *S. cerevisiae*, and thus improve xylose fermentation to ethanol. Brat et al. optimized the codon sequence of the *C. phytophermentans* XI based on the codon usage of the glycolytic pathway genes that are highly expressed in *S. cerevisiae*. This codon optimization leads to an increase of 46% in specific growth rate on xylose due to the enhanced XI activity [90]. Lee et al. used directed evolution to enhance XI activity, resulting in a mutant with 77% higher V_max_. A *S. cerevisiae* strain expressing this mutated version presented 8 times higher ethanol production and xylose consumption rate when compared to the strain engineered with the wild-type *Piromyces* sp. gene [92]. The deletion of *GRE3*, a gene that codifies for an unspecific aldose reductase involved in the formation of xylitol, also enhanced xylose assimilation and ethanol formation by *S. cerevisiae* expressing bacterial XI due to the reduction of xylitol production, which has an inhibitory effect on XI activity [93,94].

Metabolic engineered *S. cerevisiae* strains with the oxidoreductase pathway mostly express *XYL1* and *XYL2* from *S. stipitis*, coding for XR and XDH, respectively [78,95–98]. This strategy resulted in faster xylose assimilation and higher ethanol titer in comparison with strains harboring XI pathway [99,100]. Nevertheless, the XR/XDH pathway has a bottleneck caused by a cofactor imbalance between XR, which mainly uses NADPH as cofactor, and XDH, that only uses NAD^+^ to catalyze the reaction. This bottleneck especially manifests under anaerobic conditions, where NAD^+^ cannot be regenerated by the lack of oxygen. The cofactor imbalance leads to xylitol accumulation, lowering ethanol production and yield [99–101]. Several strategies were applied to overcome the redox imbalance of the XR/XDH pathway. Modify the cofactor preference of XR to NADH or XDH to NADP^+^ and combine these mutant enzymes with the wild-type allowed a reduction in xylitol formation and improved ethanol production and yield from xylose [102–105]. A similar result was obtained by replacing the *S. stipitis* XR by the NADH-preferring XR from *Sp. passalidarum* [106]. Deletion of *GRE3* also proved to be an effective way to decrease xylitol accumulation when using the XR/XDH pathway [21,107]. The cofactor imbalance can also be avoided in the presence of inhibitory compounds by increasing NADH-dependent detoxification enzymes, e.g. by expression of the *adhE* gene from *E. coli*, which encodes for an acetylating acetaldehyde dehydrogenase that reduces acetate, regenerating NAD+ for XDH activity [108,109].

Studies comparing the expression of XR/XDH and XI pathways revealed that strains with the XR/XDH pathway have higher ethanol productivity, while the XI pathway results in higher ethanol yield due to the lower formation of xylitol [99,100,110]. In the study performed by Karhumaa et al. (2007), using laboratory strains carrying XR/XDH or XI pathway, the authors attributed the higher xylose consumption of the XR/XDH-carrying strain to the higher specific activity of XR in comparison to XI, resulting in higher ethanol production, despite xylitol accumulation that leads to lower ethanol yield from xylose [99].

On the other hand, the simultaneous expression of XR/XDH and XI pathways have been attempted. Wang et al. (2017) engineered a strain capable of mixed sugar consumption, expressing both XR/XDH and XI pathways for xylose consumption. Despite a low consumption of xylose, the authors referred that the low levels of xylitol produced were the result of the expression of XI [111]. However, a comparison between sole and simultaneous expression of xylose consumption pathways is necessary to access the efficiency of XR/XDH and XI simultaneous expression. This comparison was performed by Cunha et al. (2019), where the simultaneous expression of both xylose consumption pathways in synthetic medium and in detoxified and non-detoxified corn cob hydrolyzate has been studied [112]. While in synthetic medium and detoxified corn cob hydrolyzate the XI pathway showed higher ethanol titer and yield with low xylitol production, the combination of both XR/XDH and XI pathways resulted in higher ethanol yield in non-detoxified corn cob hydrolyzate. This was the result of the cofactor equilibrium between furan detoxification (regenerating NAD+) and the XDH activity (regenerating NADH). The simultaneous expression and activity of both pathways allowed furfural and HMF detoxification and high ethanol production with low xylitol production, showing a valid strategy to increase the efficiency of ethanol production from undetoxified lignocellulosic hydrolyzates [112].

Besides the optimization of xylose consumption pathway, there are other modifications that could be applied in *S. cerevisiae* strains to improve bioethanol production, such as xylose uptake optimization. *S. cerevisiae* does not possess specific xylose transports, using native hexose transporters to assimilate xylose [113,114]. These nonspecific transporters have less affinity to xylose than glucose and show inefficient transport when xylose is present at lower concentrations, constituting a bottleneck in the development of an efficient xylose-fermenting yeast [115]. The expression of heterologous sugar transporters from native fermenting yeasts have been a successfully strategy to improve xylose transport in *S. cerevisiae*. Overexpression of *GXF1* from *Candida intermedia* increased xylose uptake rate and cell growth under low xylose concentration, while the additional expression of *S. stipitis XUT4, XUT5, XUT6, XUT7, RGT2* and *SUT4* also increased xylose uptake rate and specific growth rate in XI-harboring industrial *S. cerevisiae* [116,117]. An effective co-fermentation of glucose and xylose is desirable for bioethanol production from lignocellulosic biomass, however, xylose assimilation rate is low until the depletion of glucose. In an attempt to overcome this problem, Saitoh and collaborators (2010) constructed a high xylose assimilating yeast strain using as host a *S. cerevisiae* strain with high β-glucosidase activity on the cell surface. The β-glucosidase anchored on the surface of the yeast cell allows the conversion of cellobiose into glucose nearby the cell surface, preventing glucose accumulation outside the cell, avoiding catabolite repression. The study showed that the constructed industrial *S. cerevisiae* strain displayed complete xylose consumption in glucose/xylose and cellobiose/xylose co-fermentations, with higher xylose assimilation rate in cellobiose/xylose co-fermentation and, consequently, higher ethanol productivity [118].

### 3.2. Tolerance toward inhibitory compounds present in the hemicellulosic fraction

Tolerance to lignocellulose inhibitors is an importance feature in *S. cerevisiae* strains used for bioethanol production from the xylose enriched hemicellulosic fraction. Acetic acid, furfural, and 5-hydroxymethyl-2-furaldehyde (HMF) are the most common inhibitors that accumulate in the hemicellulosic (liquid) fraction of lignocellulose during pretreatment and/or hydrolysis, affecting yeast growth and reducing ethanol yield and productivity [119,120]. Industrial *S. cerevisiae* strains can be used to diminish this inhibitory challenge, as these robust strains display higher tolerance against stress factors like the presence of toxic compounds [20,121,122]. Additionally, several metabolic engineering strategies have been applied envisioning the feasibility of second-generation bioethanol industry (as recently reviewed by Cunha et al [14].and Brandt et al. [123]). These approaches mainly target mechanisms of (1) inhibitor detoxification, by expressing genes encoding enzymes responsible for the conversion of inhibitors into less toxic compounds [108,109,124,125] and/or by creating a redox equilibrium between the oxidoreductase xylose consumption and the detoxification pathways [108,109,124]; or (2) expression of transcription factors involved in regulation of major stress responses in *S. cerevisiae* (e.g. YAP1, HAA1) [126,127].

## 4. Consolidated bioprocessing

Consolidated bioprocessing (CBP) has been gaining increased attention to reduce the cost and increase the efficiency of bioethanol refineries, combining enzyme production, (hemi)cellulose hydrolysis and sugar fermentation into a single process by using a microorganism with both fermentation and saccharification abilities. The establishment of CBP requires the development of a robust microorganism capable of performing a simultaneous saccharification and fermentation (SSF) without addition of external enzymes. *S. cerevisiae*, being the most used microorganism for the production of bioethanol, presents outstanding fermentative performance as well as an extensive genetic toolbox for its manipulation, which makes it the ideal host to develop a CBP microorganism. Accordingly, *S. cerevisiae* has been extensively genetically modified to produce hydrolytic enzymes for the direct production of ethanol from cellulose and/or hemicellulose (Table 1).

**Table 1.** Different strategies and outcomes of consolidated bioprocessing of cellulose, hemicellulose and both, using *Saccharomyces cerevisiae* as chassis strain.β-glucosidase (BGL1), endoglucanase (EG), cellobiohydrolase I (CBHI), cellobiohydrolase II (CBHII), β-xylosidase (XylA); xylanase (Xyn); acetylxylan esterase (XynA); glucoamylase (AMG); extracellular amylase (AM); cellodextrin transporter (cdt-1); exoglucanase (EXG); arabinofuranosidase (ABF); Xylose isomerase (XI). Ethanol yield was calculated (when data were available) as the ratio of grams of ethanol produced by the total of potential fermentable sugars in the medium (for cellulose CBP only potential glucose was considered).

Strategy	Enzymes	Substrate	Ethanol titer (g/L)	Ethanol yield (g/g pot sug)	Reference
Cellulose CBP					
Secretion.	BGL1 (*Saccharomycopsis fibuligera*); EG (*Trichoderma reesei*).	10 g/L PASC	1.0	0.090	[128]
Secretion. Industrial host.	BGL1, EG (*Trichoderma viride*).	20 g/L CMC	4.6	0.21	[138]
Secretion.	BGL1 (*S. fibuligera*); EG (*Clostridium thermocellum*).	Barley straw pretreated with laccases complexes	2.3	N.D.	[153]
Secretion.	BGL1, EG (Truncated, *Fibrobacter succinogenes*); CBHII (*Chaetomium thermophilum*).	20 g/L corn cob powder	6.4	N.D.	[129]
Secretion.	Expression of a single-enzyme-system-three-cellulase gene isolated from *Ampullaria gigas* Spix. Endo-beta-1,4-glucanase, exo-beta-1,4-glucanase, and xylanase.	80 g/L of rice straw, 20 g/L of wheat bran	8.1	N.D.	[140]
Secretion.	BGL1 (*S. fibuligera*); EG (*T. reesei*); CBHI (*C. thermophilum*); CBHII (*Chrysosporiumlucknowense*).	NaOH pretreated rice straw	0	0	[130]
Secretion.	BGL1, EG, CBHI (*T. reesei*).	Alkaline peroxide pretreated wheat straw	24	0.36	[139]
Secretion. Industrial-derived host	BGL1 (*S. fibuligera*); CBHI (*Talaromyces emersonii*).	20 g/L of NaOH pretreated corn husk (44% cellulose)	3.4	0.34	[131]
		20 g/L of NaOH pretreated corn cob (43% cellulose)	3.3	0.34	
	BGL1 (*S. fibuligera*); EG (*T. reesei*).	20 g/L of NaOH pretreated corn husk (44% cellulose)	3	0.31	
		20 g/L of NaOH pretreated corn cob (43% cellulose)	4.0	0.42	
Cellulosome. Scaffoldin (*Clostridium cellulovorans*) Consortium.	BGL1 (*S. fibuligera*); chimeric EG (*Clostridium thermocellum*).	10 g/L CMC	3.4	0.31	[141]
Cellulosome. Scaffoldin (*Clostridium thermocellum*)	BGL1 (*Aspergillus aculeatus*); EG, CBHII (*T. reesei*).	10 g/L PASC	1.5	0.14	[142]
		10 g/L Avicel	1.0	0.090	
	BGL1 (*A. aculeatus*); EG, CBHII (*T. reesei*); LPMO (*Thermoascus aurantiacus*); CDH (*Humicola insolens*).	10 g/L PASC	2.7	0.24	
		10 g/L Avicel	1.8	0.16	
Cellulosome. Scaffoldin (*Clostridium thermocellum*)	BGL1, cdt-1 (*Neurospora crassa); EG (Clostridium cellulolyticum); EXG (Clostridium acetobutylicum)*.	10 g/L CMC	3.3	0.29	[144]
	BGL1, cdt-1 (*N. crassa*); *EG (Clostridium cellulovorans); CBHII (Aspergillus niger)*.	10 g/L PASC	1.1	0.10	
(Hemi)Cellulosome. Scaffoldin (C. thermocellum, Clostridium cellulolyticum and Ruminococcus flavefaciens). Consortium.	BGL1 (*A. aculeatus*); EG, CBHII (*T. reesei*).	10 g/L PASC	1.0	0.090	[157]
Cell-surface display.	BGL1 (*A. aculeatus*); EG, CBHII (*T. reesei*); AMG (*Rhizopus oryzae*); AM (*Streptococcus bovis*).	10 g/L of acid treated Avicel (8.44 g/L total sugars)	1.0	0.12	[147]
		50 g/L of cassava pulp (30% cellulose and 60% starch)	10	N.D.	
Cell-surface display. Delta cocktail integration. Diploid strain by mating.	BGL1 (*A. aculeatus*); EG, CBHII (*T. reesei*).	20 g/L PASC	7.6	0.34	[132]
		100 g/l of liquid hot water pretreated rice straw (44.8% glucan)	7.5	0.15	
Cell-surface display. Industrial host. Consortium. EG:CBHII:BGL1 ratio of 2:1:1.	BGL1 (*A. aculeatus*); EG, CBHII (*T. reesei*).	10 g/L PASC	1.8	0.16	[133]
		100 g/L steam-exploded corn stover (48.5% of cellulose)	4.0	0.074	
Cell-surface display.	BGL1 (*A. aculeatus*); EG (*T. reesei*); CBHI (*Talaromyces emersonii*); CBHII (*Chrysosporium lucknowense*).	20 g/L PASC	6.4	0.29	[152]
		10 g/L Avicel	1.6	0.15	
		100 g/L of liquid hot water pretreated rice straw (milled, 43% glucan)	1.4	0.030	
Cell-surface display. Delta cocktail integration. Expression of Artificial Zinc Finger Protein-AZFP for thermotolerance	BGL1 (*A. aculeatus*); EG, CBHII (*T. reesei*).	20 g/L PASC	8.7	0.39	[39]
		200 g/L of NaOH pretreated Jerusalem artichoke stalk	28	0.22	
Cell-surface display. Cocktail integration with ratio optimization.	BGL1 (*A. aculeatus*); EG (*T. reesei*); CBHI (*T. emersonii*); CBHII (*C. lucknowense*).	10 g/L Avicel	2.9	0.26	[151]
		25 g/L of liquid hot water pretreated rice straw (milled, 43% glucan)	0.8	0.067	
Cell-surface display. Industrial host. Oxidoreductase xylose consumption pathway.	BGL1 (*A. aculeatus*); EG, CBHII (*T. reesei*).	10 g/L PASC	2.1	0.19	[156]
		20 g/L of steam-exploded Corn Stover (48.5% cellulose)	1.2	0.11	
Hemicellulose CBP					
Secretion. Oxidoreductase xylose consumption pathway.	XylA, Xyn, ABF (*Ustilago bevomyces*)	20 g/L of xylan	0.32	N.D.	[134]
Hemicellulosome. Scaffoldin *C. thermocellum*	XylA (*A. niger*); Xyn (*T. reesei*)	10 g/L birchwood xylan	0.95	N.D.	[149]
Cell-surface display. Oxidoreductase xylose consumption pathway.	XylA (*Aspergillus oryzae*); Xyn (*T. reesei*)	Birchwood xylan corresponding to 100 g/L total sugar	7.1	0.071	[135]
Cell-surface display. Oxidoreductase xylose consumption pathway.	BGL1 (*A. aculeatus*); XylA (*A. oryzae*); Xyn (*T. reesei*)	Rice straw hemicellulose containing 26 g/L of xylose and glucose equivalents	8.2	0.32	[154]
Cell-surface display. Industrial strain. Oxidoreductase xylose consumption pathway.	BGL1 (*A. aculeatus*); XylA (*A. oryzae*); Xyn (*T. reesei*)	Rice straw hemicellulose (Liquid hot water pretreatment) containing ~24 g/L of xylose and glucose equivalents	4.0	N.D.	[124]
Cell-surface display. Isomerase xylose consumption pathway with display of *C. cellulovorans* XI. Consortium.	XylA (*A. niger*); Xyn (*Saccharophagus degradans*)	100 g/L birchwood xylan	6.0	N.D.	[136]
Cell-surface display. Industrial host. Oxidoreductase xylose consumption pathway.	XylA (*A. oryzae*); Xyn (*T. reesei*).	10 g/L birchwood xylan	1.2	N.D.	[156]
Cell-surface display. Industrial host. Oxidoreductase and isomerase xylose consumption pathways.	BGL1 (*A. aculeatus*); XylA (*A. oryzae*); Xyn (*T. reesei*)	Corn cob hemicellulosic liquor (hydrothermal pretreatment) containing 26 g/L of xylose and glucose equivalents	11	0.33	[155]
Cellulose+Hemicellulose CBP					
Cell-surface display. Industrial host. Oxidoreductase xylose consumption pathway. Consortium.	BGL1 (*A. aculeatus*); EG, CBHII, Xyn (*T. reesei*); XylA (*A. oryzae*)	20 g/L of steam-exploded corn stover (48.5% cellulose; 11.3% hemicellulose)	1.6	0.12	[156]
(Hemi)Cellulosome. Scaffoldin (Clostridium thermocellum, Clostridium cellulolyticum and Ruminococcus flavefaciens). Consortium.	BGL1 (*A. aculeatus*); EG, CBHII, Xyn (*T. reesei*); XylA (*A. oryzae*)	20 g/L of steam-exploded corn stover (48.5% cellulose; 11.3% hemicellulose)	0.92	0.069	[157]
Secretion. Industrial-derived host. Isomerase xylose consumption pathway.	BGL1 (*T. reesei*); EG (*A. oryzae*); CBHI (*T. emersonii*); CBHII (*C. lucknowense*); XylA, Xyn (*A. niger*); XynA (*Clostridium cellulovorans*)	2% (w/v) cellobiose, 2% (w/v) corn cob xylan and 2% (w/v) CMC.	< 2.0	< 0.030	[158]

**Figure 1.**
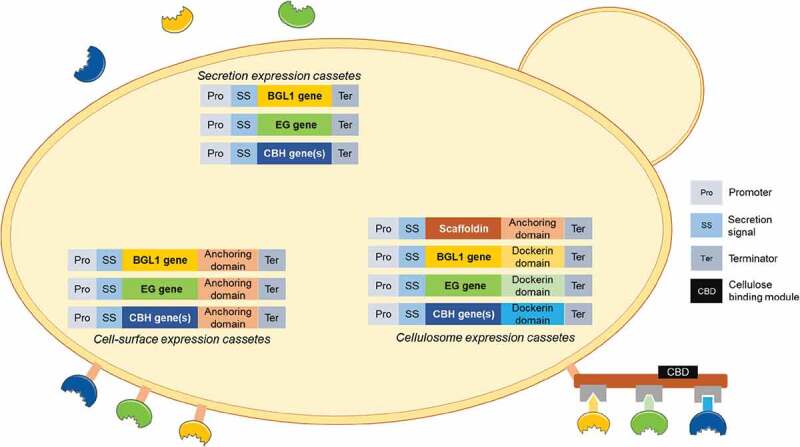
Different strategies for the production of cellulases by *Saccharomyces cerevisiae* aiming at consolidated bioprocesses.

Cellulose, containing the majority of glucose in lignocellulosic biomass and being more resistant to saccharification than hemicellulose, has been the focus for the design of a CBP hydrolytic strain. Full enzymatic hydrolysis of crystalline cellulose requires three major types of enzymatic activity: endoglucanases (EG), exoglucanases (CBH: CBH1 and CBH2) and β-glucosidases (BGL1) [137]. EG acts in the amorphous regions of cellulose generating free chain ends; CBH then hydrolyzes the reducing (CBH1) and non-reducing (CBH2) ends formed, releasing cellobiose and small cello-oligosaccharides, which are further converted into glucose by BGL1. The production of these enzymes in *S. cerevisiae* is achieved by the expression of genes from native cellulolytic microorganisms and is performed with mainly 3 different strategies: (1) secretion of enzymes, (2) assembling of a cellulosome at the cell surface, and (3) cell-surface display of enzymes (Figure 1).

On the first strategy the catalytic domain of the enzyme is fused to a secretion signal (e.g. α-factor signal peptide) to enable the yeast to secrete the heterologous enzymes into the fermentation media. With this, Gong and collaborators produced 4.6 g/L of ethanol from 20 g/L of carboxymethyl cellulose (CMC) by secreting BGL1 and EG from *Trichoderma viride* in an industrial host [138]. Also, through secretion of BGL1, EG, and CBHI from *Trichoderma reesei*, 24 g/L of ethanol were obtained from alkaline peroxide-pretreated wheat straw [139]. In a different approach, the expression of a single-enzyme-system-three-cellulase gene isolated from *Ampullaria gigas* Spix, with endo-beta-1,4-glucanase, exo-beta-1,4-glucanase, and xylanase activities, resulted in the production of 8.1 g/L of ethanol from 80 g/L of rice straw and 20 g/L of wheat bran [140].

Cellulosomes are multi-enzyme complexes, produced by anaerobic bacteria and fungi, that assemble on a noncatalytic scaffoldin protein, normally containing a cellulose-binding domain to approximate the complex to the cellulosic substrate. The cellulases, bearing specific dockerins, interact with the cohesins present in the scaffolding and form the multifunctional cellulosome. The complex is anchored to the cell wall by fusing the scaffoldin to a glycosylphosphatidylinositol (GPI) anchor, which is normally an anchoring domain selected from *S. cerevisiae* cell wall proteins (e.g., α-agglutinin AGA1 or AGA2). For the design of CBP yeast strains, the most used scaffoldins are from the *Clostridium* genera, while the cellulases are obtained from different cellulolytic organisms. Using this strategy, 3.4 g/L of ethanol were obtained from 10 g/L CMC by synthesis of a cellulosome containing the scafoldin from *Clostridium cellulovorans* and the enzymesBGL1 from *Saccharomycopsis fibuligera* and chimeric EG from *Clostridium thermocellum* [141]. In another work, the production of a cellulosome containing the enzymes BGL1 (*A. aculeatus*); EG and CBHII (*T. reesei*), lytic polysaccharide monooxygenase (LPMO, *Thermoascus aurantiacus*) and cellobiose dehydrogenases (CDH, *Humicola insolens*) assembled in a scaffolding from *C. thermocellum*, resulted in 2.7 g/L of ethanol from 10 g/L of phosphoric acid swollen cellulose (PASC) [142]. LPMOs are reported to cleave and decrystallize recalcitrant cellulose in the presence of an electron donor such as CDH, making the polymer more accessible to the activities of EG and CBH [143]. The addition of LPMO and CDH to a yeast-displayed cellulosome with BGL1, EG and CBH2 was found to increase the ethanol titers and yields from PASC and Avicel by more than 1.7-fold (Table 1), clearly showing a synergistic effect of these enzymes in degradation of cellulose. In another approach, Fan and collaborators (2016) constructed a *S. cerevisiae* strain producing a mini scafoldin with EG and CBH, as well as a cellodextrin transporter and intracellular BGL1 to decrease the product inhibition effect of glucose in the extracellular cellulases, reaching 3.3 g/L of ethanol from 10 g/L of CMC [144]. It should be noted that almost every work describing a cellulosome approach uses a consortium of several strains as a strategy to decrease the metabolic burden of the yeast host [145]: modifying a *S. cerevisiae* strain to display the scaffold of the cellulosome at its cell surface while other strains (sometimes *E. coli* strains) are used to produce the enzymes that will bind and form the cellulosome.

As for the cell surface attachment of cellulosomes, the most used method for cell surface display of enzymes is the GPI-anchoring system. For this, the target protein is fused to the anchoring domain of yeast cell wall proteins, such as α-agglutinin, SED1, or SAG1, resulting in the enzyme immobilization in the cell wall by covalent linkage to β-1,6-glucans [146]. Using this strategy, Yamada and coauthors displayed BGL1 from *A. aculeatus* and EG and CBHII from *T. reesei* in a diploid *S. cerevisiae*, producing 7.6 g/L and 7.5 g/L of ethanol from 20 g/L of PASC and 100 g/l of liquid hot water pretreated rice straw, respectively. The display of these cellulases in addition to a glucoamylase (AMG; *Rhizopus oryzae*) and extracellular amylase (AM; *Streptococcus bovis*) allowed the production of 10 g/L of ethanol from 50 g/L of cassava pulp, a low-cost byproduct of starch industry containing 30% cellulose and 60% starch [147].

Both cellulosome and cell surface display strategies benefit saccharification due to the proximity between the different enzymes which allows them to work synergistically on the substrate [148,149]. Besides, the proximity of the enzymes to the cell wall is also advantageous, as the liberated sugar monomers can be readily assimilated by the yeast. The ratio of the different enzymes may also affect the hydrolytic capacity of the yeast. Taking this into account, a cocktail δ-integration method was developed, where multiple copies of the genes encoding the different cellulases are simultaneously integrated in theδ sequences of *S. cerevisiae* followed by a screening to select the strain with higher cellulolytic activity (i.e. with an optimum ratio of cellulases) [150]. Using this method, Liu and coauthors (2017) constructed a yeast displaying an optimized ratio of BGL1 (*A. aculeatus*), EG (*T. reesei*), CBHI (*Talaromyces emersonii*) and CBHII (*Chrysosporium lucknowense*), increasing in 60% the ethanol titer from liquid hot water pretreated rice straw, in comparison with a strain with single copy integration of each of the enzyme encoding genes [151].The optimal ratio is expected to differ depending on the crystallinity of the cellulosic substrate and should be optimized for each type of lignocellulosic biomass and pretreatment method.

Temperature, as for SSF processes, is also a key determinant for CBP efficiency, as the optimal temperature for the activity of the hydrolytic enzymes is higher than the optimal for yeast fermentation. In fact, the highest reported titer of ethanol resulting of CBP uses an *S. cerevisiae* host producing not only cellulases, but also an artificial zinc finger protein to increase its thermotolerance, which allowed the CBP to be performed at 42°C resulting in the production of 28 g/L of ethanol from NaOH-pretreated Jerusalem artichoke stalk [39]. Taking this into account, the selection of industrial *S. cerevisiae* strains which have shown capacity to ferment at higher temperatures [21,112], may be an advantage to develop CBP microorganisms, decreasing the need for further genetic engineering besides hydrolases production and consequently the risk of metabolic burden.

The hydrolysis efficiency is dependent not only on the yeast host and enzymes’ source, ratio and localization strategy, but also on the types of substrates. When comparing the same strain(s) and strategy in different substrates it is clear that the hydrolysis capacity increases with the decrease of crystallinity index of the substrate: the ethanol yield from PASC is higher than from Avicel [142,152] and the ethanol yield from CMC is higher than from PASC [144]. This highlights the importance of the pretreatment method of lignocellulose: it should be harsh enough to decrease the biomass recalcitrance and decrease its crystallinity but should not extensively degrade its components (e.g., sugars into furanic compounds or lignin into phenolic compounds). From the works describing conversion of real lignocellulosic biomass, the ones using alkali pretreatments obtained higher ethanol yields, even though there is a lack of a direct comparison study (Table 1). Also, Hyeon and collaborators used an enzymatic pretreatment with laccase complexes to delignify barley straw and produced 2.34 g/L of ethanol with a *S. cerevisiae* strain secreting endoglucanase and β-glucosidase [153]. However, from the point of view of an integrated biorefinery, with recovery and utilization of the various fractions of lignocellulosic, these methods are disadvantageous due to the high degradation of hemicellulose and lignin.

Another important factor for valorization of lignocellulosic biomass through CBP is the utilization of hemicellulose, and some works focused on the production of ethanol solely from this fraction (Table 1). After pretreatment, inhibitory compounds accumulate in the liquid (hemicellulosic) fraction, and the robust traits of industrial *S. cerevisiae* isolates may once more be an advantage. Hemicellulose main component is xylan, and its degradation requires the activity of xylanases (Xyn) to cleave the major chain into small xylooligosaccharides which are then hydrolyzed into xylose by xylosidases (XylA). Small amounts of cellobiose and small cello-oligosaccharide are also present in the hemicellulosic fraction, so the production of BGL1 is also desirable to convert them into fermentable glucose. It should be noted that, to directly produce ethanol from hemicellulosic material the hemicellulase-producing *S. cerevisiae* strain also needs to be engineered to be capable of xylose consumption, and the approach used (oxidoreductase vs isomerase pathways) can also highly influence its fermentative performance [112]. Cell surface display of enzymes is the most used strategy for xylan degradation, with the reported secretion and cellulosomes approaches resulting in productions of less than 1 g/L of ethanol (Table 1). Sakamoto and collaborators displayed BGL1 (*A. aculeatus*), XylA (*Aspergillus oryzae*), and Xyn (*T. reesei*) in the cell surface of a strain containing the oxidoreductase xylose pathway, resulting in the production of 8.2 g/L of ethanol from rice straw hemicellulose [154]. The display of the same hemicellulases on the cell surface of an industrial strain containing both isomerase and oxidoreductase xylose consumption pathways resulted in 11 g/L of ethanol from a corn cob hemicellulosic liquor [155].

Recently, works have focused in CBP of both cellulose and hemicellulose: Chen and collaborators combined a cellulase-displaying strain with a hemicellulase-displaying strain to produce 1.6 g/L of ethanol from 20 g/L of steam-exploded corn stover (48.5% cellulose, 11.3% hemicellulose) [156]; Tian and collaborators used a consortium of modified *S. cerevisiae* to construct a yeast displaying a (hemi)cellulosome, which produced 0.92 g/L of ethanol from steam-exploded corn stover (48.5% cellulose, 11.3% hemicellulose) [157]; Claes et al. used a robust industrial-derived strain to secret both cellulases and hemicellulases and produced less than 2 g/L of ethanol from a mixture of 2% (w/v) cellobiose, 2% (w/v) corncob xylan and 2% (w/v) CMC [158]. While desirable for the goal of CBP, the design of a yeast with capacity to hydrolyze both cellulose and hemicellulose requires extensive modifications and requires further optimizations to prevent metabolic burden and achieve feasible ethanol titers and yield.

## 5. Concluding remarks

*S. cerevisiae* is the preferred organism for bioethanol production, and several genetic engineering strategies have been developed aiming at the economic viability of second generation bioethanol plants: improving the valorization of lignocellulosic biomass through consumption of its major sugars, preventing extra costs by endogenous producing hydrolytic enzymes, and an overall easing of the process conditions by improving traits such as thermotolerance, flocculation, and tolerance toward inhibitory compounds. Also, *S. cerevisiae* strains isolated from industrial harsh conditions have proved to be an important source of chassis to be explored, due to their higher robustness and innate particularities. Furthermore, the extensive knowledge on *S. cerevisiae* metabolism and response to lignocellulosic biomasses, resulting from the studies of second generation bioethanol, together with the existing genetic toolbox facilitates the use of this yeast as a microbial cell factory to produce a plethora of high-value chemicals, supporting both the energetic and economic goals of a biorefinery. Nonetheless, and despite the advances made in the last years, research still needs to focus on the development of an engineered *S. cerevisiae* strain that could be applied and sustain a CBP bioethanol production plant. In fact, and regardless of the low ethanol titers that are still obtained with a purely CBP process, the developed CBP strains can also be applied in the industrial processes with the objective of reducing the quantity requirements for exogenous enzymes. This has already been performed in first-generation processes, where the use of an engineered starch-hydrolyzing *S. cerevisiae* strain decreased the use of exogenous enzymes by more than 50% [159]. Nevertheless, and as it is generally recognized, the economic feasibility of second generation bioethanol is dependent on the production of ethanol titers superior to 4% (w/w), to diminish the energy demand in the distillation stage [160,161]. At this moment, the maximum ethanol titer obtained from a CBP process with an engineered *S. cerevisiae* strain is 28 g/L, which is still far from the critical threshold (~40 g/L of ethanol). In order to reach that goal with *S. cerevisiae*, combined efforts are needed, such as: (1) improvements of pretreatment technology to make cellulose more accessible to enzymatic hydrolysis; (2) supplementation with additional low-value carbon sources to increase ethanol concentration; (3) enzyme engineering and/or prospecting to allow the development of tailor made proteins for CBP processes (e.g. with higher hydrolytic activity at lower temperatures); (4) development of even more thermotolerant strains that would allow the process to occur at higher temperatures to favor hydrolysis without compromising fermentation (the highest temperature described for a CBP process with *S. cerevisiae* is 42ºC, which also represents the higher ethanol titer of 28 g/L obtained so far with CBP); (5) construction of *S. cerevisiae* strains capable of an efficient xylose consumption and tolerance toward lignocellulose-derived inhibitors, which would allow a real valorization of the hemicellulosic fraction, which can make an actual difference in terms of ethanol titers (CBP of hemicellulose with xylose-fermenting *S. cerevisiae* have resulted in the production of ethanol concentrations of up to 11 g/L); and (6) optimization of the expression system for production of hydrolases by *S. cerevisiae*, allowing higher enzyme quantities and degradation efficiency. Altogether, these advances would allow an efficient consolidated process for second generation bioethanol plants using *S. cerevisiae* strains, completely removing the cost of exogenous enzymes and surpassing the current barrier of limited ethanol titers.

## Highlights

- Genetic engineering strategies to improve second generation ethanol production
- Industrial strains with potential traits for application in lignocellulosic processes
- *Saccharomyces cerevisiae* as a key microorganism for lignocellulosic biorefineries
